# Why Workers Hesitate to Report Their Work-Related Musculoskeletal Symptoms: A Survey at a Korean Semiconductor Company

**DOI:** 10.3390/ijerph182111221

**Published:** 2021-10-26

**Authors:** Jong-Tae Park, Jangwhon Yoon

**Affiliations:** 1 Department of Occupational and Environmental Medicine, Korea University Ansan Hospital, Ansan 15355, Korea; impjt@korea.ac.kr; 2 Department of Physical Therapy, Hoseo University, Asan 31499, Korea

**Keywords:** occupational accident, work-related musculoskeletal disorders, underreporting, surveillance

## Abstract

Underreporting work-related musculoskeletal disorders (WRMSD) has been an issue in South Korea. The purpose of this survey was to figure out how many employees of a semiconductor and liquid crystal display company in South Korea experience WRMSDs and what the possible obstacles in reporting to the company are. A survey was developed with demographic questions, perceived WRMSD symptoms, and reasons for reporting or not reporting WRMSD. The survey was distributed via the company’s intranet to all employees (24,380) whose employee identification number ended with an odd number. A total of 2862 employees completed the survey and the response rate was 11.7%. A total of 55.2% of respondents had felt at least one musculoskeletal symptom during the past year. More than 40% of workers who had experienced pain or discomfort during the past year thought their symptoms were more than 50%. work-related. More than one-fourth of respondents answered that they did not report their symptoms to the company more than once. The open-ended answers for not reporting WRMSD were categorized into seven common reasons. The reasons for not reporting WRMSD in previous studies show a combination of personal, organizational, socioeconomical, and cultural factors. To encourage and manage WRMSD effectively, seven recommendations of authors are described.

## 1. Introduction

Underreporting work-related musculoskeletal disorders (WRMSD) has been a long-lasting occupational health issue in South Korea. There must be complex interaction among personal, organizational, socioeconomical and cultural concerns in reporting WRMSD. The non-fatal injury rate in Korea was one-fifth of the average in EU15 countries, while the fatal injury rate was five times higher [1]. This phenomenon of high fatal and low non-fatal injury rates was found in occupational injury statistics in other Asian countries like Japan, Taiwan, and Singapore [1]. Fatal occupational injuries in construction industry are suggested to be interpreted with caution due to underreporting and the different surveillance system in China [2]. In Japan, only 22% of staff who had experienced needlestick injuries in operating rooms reported their incident (Nagao et al., 2009). Accurate reporting of work-related conditions was reinforced to monitor workplace health and safety, and to identify the most needed interventions [3].

In contrast to Western countries, of 18,725,160 workers working at 2,680,874 workplaces that are eligible for workers’ compensation in 2019, 109,242 workers had occupational accidents, and so the occupational accident rate was 0.58% in Korea [4]. They needed at least four or more days of medical care (does not necessarily mean days away from work) to apply. This was 0.70% in 2009 [1]. In the US, the incidence rate of employer-reported workplace injuries and illness in private industry was 2.8 (2.80%) cases per 100 fulltime equivalent workers and the severe nonfatal injuries and illnesses based on the days away from work was 0.87% in 2019 [5]. Across the EU-27, there were 1769 (1.77%) non-fatal accidents per 100,000 persons employed in 2018 [6]. A low occupational accident rate does not mean there are fewer musculoskeletal disorders in Korea. Regardless of its work relatedness, four of the top ten diagnoses for hospitalization were musculoskeletal problems and lower back pain is the seventh most common reason for visiting medical doctors in 2019 according to the Korean Health Insurance Review and Assessment Service [7].

This company is a world-leading semiconductor, display, and smartphone manufacturer. Their annual income is about 17% of the GDP of South Korea [8]. The employees are at the highest level of education, benefit, welfare, and salary in South Korea. There have been zero WRMSD reports and no workers’ compensation claims since 2016 (merely one cardiopulmonary problem). The purpose of this survey was to figure out how many employees of a semiconductor and liquid crystal display (LCD) company in South Korea experience WRMSDs and what the possible obstacles in reporting to the company are. The survey was a part (Team 3) of the Ombudsperson Commission [9], a third-party investigation (a rare opportunity!) to evaluate the employees’ health management system and to improve the health promotion program.

## 2. Materials and Methods

### 2.1. Occupational Health Management Program of the Ombudsperson Commission

The Ombudsperson Commission was established with the nominated advisory committee members in five areas of expertise as an external independent organization to carry out inspections of the occupational health management system of the company (Semiconductor and LCD divisions) and conduct research on employees’ health from 1 November 2016 to 31 December 2017. The specific research topics of each expert were as follows. The current study was a part of the third team.

1. The physical and chemical materials team conducted studies of the overall evaluation of physical/chemical factors and radiation in the workplaces;
2. The health effect evaluation team performed research entitled ‘Health effect evaluation and the prevention of injuries to semiconductor industry workers: Establishing a cohort study and preliminary investigation’;
3. The reinforcement of health management system team studied the diagnosis and improvement plan of health-promotion activities, a comprehensive evaluation of the healthcare management system;
4. The survey and research team conducted studies of future strategies of workplace safety and health environment for occupational accident prevention;
5. The regulation team performed research on the communication of information on chemical substances and the storage of material safety data sheets.

### 2.2. Survey Development

Because of underreporting trends in WRMSD symptoms, worker surveys and symptom reports can provide more valuable and timely information than the injury records of a company or a government agency [3]. No validated survey addresses the purpose of the current study to the authors’ knowledge; therefore, a survey was developed with demographic questions, WRMSD symptoms, reporting experience, and reasons for not reporting WRMSD reasons when applicable. Demographic questions were for age, gender, type of job, job position (level 1 to 4, where level 1 is the lowest in the hierarchy), job schedule, monthly income, level of education, and marriage status. “Have you felt any pain or discomfort (aching, tightness, soreness, tingling, etc.) in any part of your body (neck, shoulder, elbow, hand, lower back, and foot) during the past year?” was asked and the part of body was specified, allowing multiple answers. The workers were asked if they had experienced pain or discomfort in any part of body, and how much (less than 10%, 30%, 50%, 70%, or more than 90%) of their symptom was perceived as ‘work-related’. They were also asked whether the symptom was reported to the supervisor or the employee health management team at the department of human resources, and, if not reported, how many times it happened and the reason were asked separately. The reason for not reporting was asked in an open-ended, multiple-choice fashion, with an ‘other’ option provided for writing down their own reasons. The answers for not reporting WRMSD were manually categorized into seven common reasons. These questions were part of a larger questionnaire including social history, annual physical assessment, on-campus medical clinic participation, health promotion programs, work/life balance, and burnout symptoms (those are not included in this study).

### 2.3. Survey Dissemination

The survey was undertaken via the company’s intranet for 13 days from 10 October to 22 October 2017. The questionnaire was distributed to all employees (24,380) whose employee identification number ended with an odd number. The purpose and procedure of the survey was explained and a consent form (IRB of Korea University Medical Center No. AS17108/001-003) was signed prior to opening the questionnaire page. There was no direct benefit for participation and the survey was totally anonymous.

### 2.4. Statistical Analysis

The characteristics of respondents were presented using descriptive statistics. To compare the prevalence of musculoskeletal symptoms, the chi-squared test was used. Responses were organized in Microsoft Excel (Microsoft Corporation, Redmond, Washington, DC, USA) and were analyzed using IBM SPSS Statistics version 24 (IBM Corp., Armonk, NY, USA). The statistical significance was set at *p* < 0.05 with a two-tailed test.

## 3. Results

### 3.1. Respondents

The questionnaire was distributed to all employees (24,380) whose employee identification number ended with odd numbers. A total of 2862 employees completed the survey (response rate: 11.7%). The characteristics of the respondents are summarized in Table 1. No answer was not included in the calculation of percentage.

The mean age of respondents was 33.1 ± 7.5 (ranging from 18 to 59) years and about 80% were in their 20s and 30s. This was similar to the mean age (33.9 ± 6.2) of all employees reported by the Department of Health Management. There were more male (69.5%), manufacturing (52.0%), job level 2 (44.6%), day duty (64.1%), monthly income ranging from $2500 to $4200 (46.5%), 4-year college graduates (36.2%), and married (56.6%) respondents.

### 3.2. Musculoskeletal Symptoms and Subjective Level of Work-Relatedness

A total of 1580 (55.2%) of the 2862 respondents felt at least one musculoskeletal symptom during the past year (Table 2). The prevalence of musculoskeletal symptoms was calculated from the frequency divided by the respondents. The prevalence of musculoskeletal symptoms was significantly greater in female (74.0%), manufacturing job (58.5%), job level 1 (62.8%), shift duty (61.7%), and high school graduates (63.5%). There was no significant difference in age, monthly income, and marriage status. When multiple answers were allowed, a musculoskeletal symptom in the neck was most frequent (40.0%), followed by the shoulder (38.6%), lower back (31.2%), hand and wrist (24.3%), leg and foot (17.8%), and arm and elbow (9.7%).

When respondents answered that they had experienced pain or discomfort in any part of body during the past year, they were asked how much of their symptom was subjectively considered ‘work-related’ or ‘job contributed’. The perceived work-relatedness of their musculoskeletal symptom was greatest at less than 10% (30.1%), and followed by 30% (28.6%), 50% (21.7%), 70% (11.6%), and more than 90% (8.0%). If 50% is the border line to be considered as WRMSD, about 41.3% of the respondents thought themselves to have work-related musculoskeletal symptoms.

### 3.3. Reasons for Not Reporting

**Figure 1:**
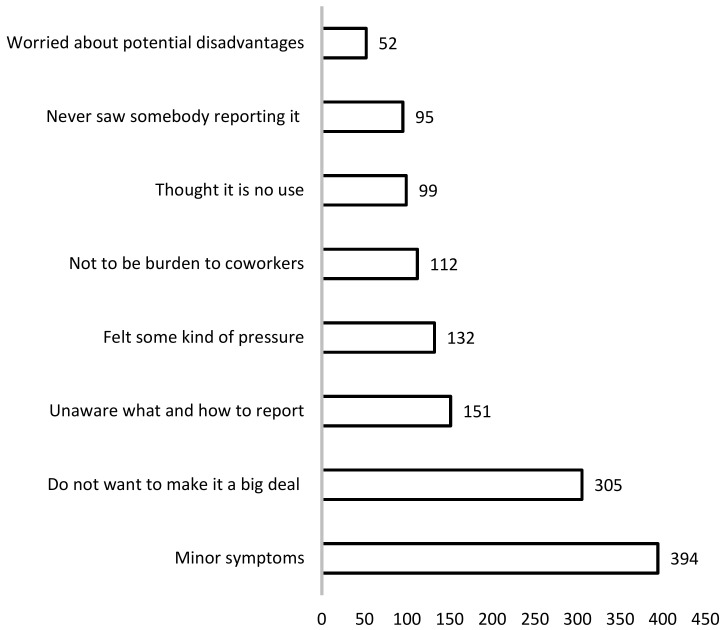
The reasons for not reporting WRMSD to the company (allowed multiple answers).

The workers were asked “How many times you did NOT report the musculoskeletal symptoms to the supervisor or the employee health management team at the department of human resource.” A total of 776 (49.1%) of all 1580 respondents with WRMSD symptoms answered that they did not report their symptoms to the company more than once. A total of 189 (12.0%) did not report two to three times and 200 (12.7%) did not more than four times since they had started to work at the company. The reasons (Figure 1) why they did not report were solicited, with multiple answers allowed. The most frequent answer was because (1) The symptoms were not serious and they recovered soon (394 respondents); followed by (2) They did not want to make it a big deal for their department and supervisors (305); (3) They did not know what and how to report (151); (4) They felt some kind of pressure in reporting the symptom (132); (5) They did not want to be a burden for their co-workers (112); (6) They thought that reporting to the company is of no use (99); (7) They had never seen somebody reporting it (95); and (8) They worried about potential disadvantages in the future (52). The number of valid responses was 1340 answers from 573 respondents.

## 4. Discussion

The purpose of this survey was to figure out how many employees at a semiconductor and LCD company in South Korea experienced WRMSDs and what the possible obstacles in reporting to the company are. The response rate was 11.7%. A total of 55.2% of respondents felt at least one musculoskeletal symptom during the past year. More than one-fourth (27.4%) of respondents answered that they did not report their symptoms to the company more than once.

In most occupational epidemiologic studies, low back pain is usually the most affected body region, but neck pain was the most frequent in the study sample. We think the type of work they do (mainly computer work with no considerable physical demand) is the reason for this difference. The prevalence of musculoskeletal symptoms in this study was significantly greater in female (74.0%), manufacturing job (58.5%), job level 1 (62.8%), shift duty (61.7%), and high school graduates (63.5%). The WRMSDs in Korea based on workers’ compensation statistics has significantly increased since the non-accidental low back pain was included in 2000 and accidental low back pain was included in 2006 as a type of WMSD [10].

### 4.1. Underreporting

Reporting WRMSD is not very easy in South Korea. To be compensated, the worker needs to report an occupational injury (including an occupational disease) and to prove its work-relatedness. Occupational injuries in Korea require more than 3 days of medical treatment to be accepted for workers’ compensation, while 1 to 3 days of absence is needed in most European countries [11]. Furthermore, the claiming worker is required by its work-relativeness to be compensated. In addition to the hurdle in the injury reporting system, personal, organizational, socioeconomical, and cultural factors interact in the decision-making process of reporting WRMSD. Workers hesitate to report, supervisors ignore, companies hide, and government agencies try to minimize WRMSDs. A report of the United States Government Accountability Office [12] concluded that workers, employers, and health practitioners all experience pressure to avoid reporting WRMSD; workers’ fear of disciplinary action and safety incentive programs that reward low injury rates are common disincentives for reporting. The Korean social support system is improving but it is not yet sufficient to encourage reporting WRMSD symptoms when the symptoms are not serious. Social perspectives on work-related disability are still not very friendly or sympathetic in the general public.

Previous studies have found that up to 81% of work-related injuries and illnesses were unreported by employees [3,13,14,15,16,17,18]. In this survey (not included in the result of this study), 444 (16%) of respondents answered that they had at least one day off due to musculoskeletal symptoms during the past year, while 1016 (35.5%) respondents answered that they had at least one day off due to general health problems including the common cold, headache, stomach ache, menstruation pain, etc. A total of 653 respondents (41.3%) of the 1580 who experienced pain or discomfort in any part of body during the past year thought their symptoms were more than 50% work-related. However, nobody claimed the workers’ compensation for their musculoskeletal symptoms during this period of time. Underreporting was more common among the white-collar workers, including those with office and research jobs. However, according to the company’s official record, only 2–3% of employees used sick days regardless of the type of symptoms and the mean duration of sick days was 38.1 ± 9.2 days for the last 5 years. As Nagao et al. [19] cautioned, the underestimation of WRMSD due to underreporting may lead to inaccurate information regarding the overall risk of exposure, and delay improvements in future prevention.

### 4.2. Reasons for Underreporting

Previous studies have shown that the reasons for not reporting WRMSD symptoms involve a combination of personal, organizational, socioeconomical, and cultural factors. Pransky and his colleagues [3] described several reasons for not reporting injuries in the US, including fear of reprisal, a belief that pain was an ordinary consequence of work activity or ageing, lack of management responsiveness after prior reports, and a desire not to lose their usual job. Probst et al. [14] found that organizations with a poor safety climate had significantly higher rates of underreporting (81% of eligible injuries unreported) compared with organizations with a positive safety climate (47% of eligible injuries unreported). In Italy and the US, as job insecurity increased, the underreporting of accidents increased [13]. Galizzi et al. [17] found that applying for workers’ compensation could lead to time pressure and workers’ doubts about eligibility, reputation, income loss, and career prospects (discrimination, missed promotions, or job loss) in the US health care industry. In the US Army [20], the most common reasons for not reporting injuries (49% of all injuries were not reported to medical personnel) were fear that an injury might affect future career opportunities and avoidance of military “profiles” (mandated physical restrictions). Tucker and his colleagues [21] suggested the reasons for not reporting may be learned and practiced early in one’s working career.

Studies [16,21] have consistently found that injury severity is strongly related to claim submission as in this study. Although variance exists across jurisdictions, the government agency must keep records of all work-related injuries and separately report more severe work-related injuries as in the US [5]. Azaroff and his colleagues [22] classified nine conceptual filters to understand the reasons for underreporting by the Bureau of Labor Statistics, including workers’ compensation wage-replacement documents, physician reporting systems, and medical records of treatment being charged to workers’ compensation.

### 4.3. Suggestions to Encourage Reporting the WRMSD

In 2016, the on-campus medical clinic at the company was already managing the 445 ‘pain complainers,’ based on the NIOSH classification: complaining of severe or very severe musculoskeletal pain lasting longer than 1 week, at least once a month for 1 year. This is much less than the results of the survey in this study. To manage WRMSD effectively and to promote employees’ health, these are the recommendations of the authors of this study:

1. Define what constitutes a reportable occupational injury and/or disorder clearly. A broader definition of WRMSD, with less concern whether it is work-related or not, would encourage early reporting and enable more effective management;
2. Reinforce the responsibility of supervisors or department heads in surveillance. If there are too many or too few incidences in a certain department, there needs to be a thorough investigation to figure out the cause of the problem;
3. Train the employees, supervisors, and employer in what, why, when, and how to report WRMSD to encourage reporting even when the symptoms are not serious enough for medical care;
4. Reassure the individuals and supervisors who report an injury/disorder that they will not be penalized. It is important for employers to demonstrate that they are open to reports of injuries [21];
5. Record all the cases even when they are not so serious and use the data to prevent WRMSD in the future. WRMSD is much easier to manage when the symptoms are mild;
6. Follow up and reward the action taken to correct the problem, not just a low injury rate.

The safety incentive programs of a company typically reward supervisors and employees for reducing workplace injury rates, but unintentionally inhibit proper reporting [3]. Safety incentive programs should be carefully designed not to encourage underreporting.

### 4.4. Limitations and Implications

This was a survey in a single electronics company, the biggest and most profitable in South Korea. Another division of the Ombudsperson Commission found 94% job satisfaction overall. The findings of this study cannot be representative of Korean industries. The questionnaire was totally anonymous; however, the respondents might have felt some kind of pressure since it was distributed via the company’s intranet instead of a third-party survey tool, such as Survey Monkey^tm^ (SurveyMonkey, San Mateo, CA, USA) and Google Forms^tm^ (Google, Mountain View, CA, USA). The survey of this study was done for 13 days only because it was a part of the complete survey of the Ombudsperson Commission. Each part of the Committee had their own survey questionnaires and schedules. With a longer period of data collection, there could potentially be a beneficial increase in response rate. The response rate of this study was 11.7%, which means it does not necessarily represent the true prevalence of WRMSD symptoms at the company. However, this response rate of 11.7% was better than usual (the company intranet allows an enormous survey dissemination on regular basis), according to the human resources representative of the company. In addition, the survey asked for the subjective symptoms, not established musculoskeletal disorders in particular. Further research needs to be undertaken at a national level to help elucidate the extent of underreporting of WRMSD among Korean companies in various economic fields.

## 5. Conclusions

Preventing WRMSD and improving productivity are not two different things. To manage WRMSD effectively, early detection and intervention is very important. The findings of this survey suggest that the surveillance system for WRMSD symptoms has to be more specific and it needs to be clearly announced that employees and employers need not worry about reporting WRMSD symptoms. Early reporting of WRMSD symptoms is good for everybody.

## Figures and Tables

**Table 1:** Characteristics of the respondents (*n* = 2862).

Characteristics	Description	Respondents	Percentage (%)
Age (*n* = 2683)	18–19	48	1.8
	20–29	953	35.5
	30–39	1169	43.6
	40–49	448	16.7
	50 and above ^1^	65	2.4
Gender (*n* = 2831)	Male	1968	69.5
	Female	863	30.5
Type of job (*n* = 2822)	Manufacturing job	1466	52.0
	Office job	424	15.0
	Research job	932	33.0
Job position (*n* = 2652)	Level 1	570	21.5
	Level 2	1183	44.6
	Level 3	655	24.7
	Level 4	244	9.2
Job schedule (*n* = 2729)	Day duty	1749	64.1
	Shift duty	980	35.9
Monthly income (*n* = 2744)	Less than $2500	772	28.1
	$2500–$4200	1275	46.5
	$4200–$6700	564	20.6
	More than $6700	133	4.8
Level of education (*n* = 2702)	High school	814	30.1
	2-year college	569	21.1
	4-year college	977	36.2
	Graduate school	342	12.7
Marriage status (*n* = 2710)	Married	1533	56.6
	Unmarried	1177	43.4

^1^ Employees retire at 60.

**Table 2:** Frequency and prevalence (frequency/respondents) of musculoskeletal symptoms experienced during the past year (*n* = 1580).

Characteristics	Description	Frequency	Prevalence
Age (*n* = 2683)	18–19	21	43.8%
	20–29	546	57.3%
	30–39	656	56.1%
	40–49	229	51.1%
	50 and above ^1^	39	60.0%
Gender * (*n* = 2831)	Male	927	47.1%
	Female	639	74.0%
Type of job * (*n* = 2822)	Manufacturing job	858	58.5%
	Office job	220	51.9%
	Research job	484	51.9%
Job position * (*n* = 2652)	Level 1	358	62.8%
	Level 2	653	55.2%
	Level 3	342	52.2%
	Level 4	121	49.6%
Job schedule * (*n* = 2729)	Day duty	907	51.9%
	Shift duty	605	61.7%
Monthly income (*n* = 2744)	Less than $2500	450	58.3%
	$2500–$4200	671	52.6%
	$4200–$6700	321	56.9%
	More than $6700	79	59.4%
Level of education * (*n* = 2702)	High school	517	63.5%
	2-year college	335	58.9%
	4-year college	463	47.4%
	Graduate school	178	52.0%
Marriage status (*n* = 2710)	Married	850	55.4%
	Unmarried	655	55.6%

^1^ Employees retire at 60. *: *p* < 0.05.
